# Automated Identification and Location Analysis of Marked Stem Cells Colonies in Optical Microscopy Images

**DOI:** 10.1371/journal.pone.0080776

**Published:** 2013-12-09

**Authors:** Vincenzo Paduano, Daniela Tagliaferri, Geppino Falco, Michele Ceccarelli

**Affiliations:** 1 Bioinformatics Lab, Genetic Research Institute “G. Salvatore” (IRGS) c/o BioGeM s.c.a r.l., Ariano Irpino, Avellino, Italy; 2 Stem Cell Research Lab, Genetic Research Institute “G. Salvatore” (IRGS) c/o BioGeM s.c.a r.l., c.da Camporeale, Ariano Irpino, Avellino, Italy; 3 Department of Science and Technologies, University of Sannio, via Port'Arsa, Benevento, Benevento, Italy; Baylor College of Medicine, United States of America

## Abstract

Embryonic stem cells (ESCs) are characterized by two remarkable peculiarities: the capacity to propagate as undifferentiated cells (*self-renewal*) and the ability to differentiate in ectoderm, endoderm, and mesoderm derivatives (*pluripotency*). Although the majority of ESCs divide without losing the pluripotency, it has become evident that ESC cultures consists of multiple cell populations highlighted by the expression of early germ lineage markers during spontaneous differentiation. Hence, the identification and characterization of ESCs subpopulations represents an efficient approach to improve the comprehension of correlation between gene expression and cell specification status. To study markers of ESCs heterogeneity, we developed an analysis pipeline which can automatically process images of stem cell colonies in optical microscopy. The question we try to address is to find out the statistically significant preferred locations of the marked cells. We tested our algorithm on a set of images of stem cell colonies to analyze the expression pattern of the *Zscan4* gene, which was an elite candidate gene to be studied because it is specifically expressed in subpopulation of ESCs. To validate the proposed method we analyzed the behavior of control genes whose pattern had been associated to biological status such as differentiation (*EndoA*), pluripotency (*Pou5f1*), and pluripotency fluctuation (*Nanog*). We found that *Zscan4* is not uniformly expressed inside a stem cell colony, and that it tends to be expressed towards the center of the colony, moreover cells expressing *Zscan4* cluster each other. This is of significant importance because it allows us to hypothesize a biological status where the cells expressing *Zscan4* are preferably associated to the inner of colonies suggesting pluripotent cell status features, and the clustering between themselves suggests either a colony paracrine effect or an early phase of cell specification through proliferation. Also, the analysis on the control genes showed that they behave as expected.

## Introduction

Over the past few years it has become evident that *in vitro* mouse ESC cultures consist of multiple cell populations [1] with different degrees of pluripotency [2], [3]. The culture heterogeneity is mainly to be addressed to ESC responsiveness to paracrine effects and cell-to-cell interaction. This colony-relative cell position analysis may result very useful to set up biological hypotheses that may lead to the understanding of cell cycle, cell differentiation, and cell meta-stable status, following the location pattern inside the colony itself. Due to the amount of images that can be collected with actual imaging technologies and the subjectivity of manual image annotations, the development of automated high throughput image annotation pipelines is an active research topic in computational biology [4]–[7].

**Figure 1:**
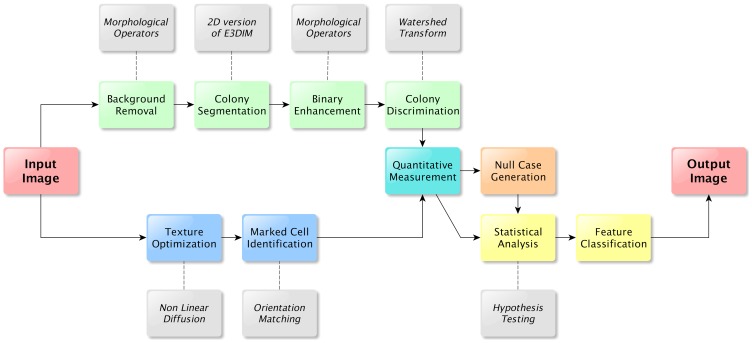
Process flow diagram for the proposed approach. A preprocessing step is used to remove the background and uniform the light intensity conditions, then the segmentation process takes place with a two-dimensional version of the Enhanced Interaction Model. The resulting binary image is then enhanced through a cascade of morphological operators. Different colonies are then processed through a Watershed transform which returns as output the segmentation for each colony in the image.

In order to monitor ESCs containing reporter genes which are markers of ESC heterogeneity we developed an analysis pipeline which can automatically process images of stem cell colonies in optical microscopy. In our pipeline the colonies are first segmented and the marked cells are then identified with an adapted filter [8] based on Orientation Matching [9]. Thereafter, quantitative information is extracted and statistical analyses are then performed on the collected data in order to find out the preferred location of the marked cells and if there is a statistically significant difference with respect to a specific *null* model. The overall pipeline of our procedure is depicted in Figure 1, where each step is detailed in Materials and Methods.

Since heterogeneous expression is traditionally associated to early cell fate decision occurring spontaneously in ESCs, we used the developed pipeline to analyze the location of cells expressing the gene *Zscan4* within ESC colonies. *Zscan4* [10] is a crucial factor, responsible for maintaining chromosomal stability, it is expressed heterogeneously in the conventional culture of murine ESCs and as it is involved in telomere elongation [11]. Our pipeline shows that cells expressing *Zscan4* are not uniformly located, rather they tend to localize near the colony center, which suggests — we hypothesize — pluripotent cell status features. Moreover the discovery that the cells expressing *Zscan4* cluster between themselves manifests a typical specification action of these cells. In addition, as a validation of the developed method, we consider as “control genes” *EndoA*, *Pou5f1*, and *Nanog*, whose location pattern can be predicted by previous studies. *Pou5f1*, a marker of pluripotency, is expressed in undifferentiated ESCs in the center of ESC colonies, with reduction or absence of expression at the more differentiated, epithelioid edges of colonies and isolated cells; *EndoA*, a marker of trophectoderm and visceral endoderm, is detected in the flatter cells that surround undifferentiated colonies whereas *Nanog* expression was rather heterogeneous compared to *Pou5f1*.

## Materials and Methods

### Culture Preparation

The mouse ES parental cell line E14Tg2a.4 derived from strain 129P2/OlaHsd [12] was cultured for two passages on gelatin–coated feeder–free plates and subsequently maintained in gelatin–coated six–well plates in complete ES medium: DMEM (Dulbeccos Modified Eagles Medium, *EuroClone*), 15% FBS (*EuroClone*), 1000 U ml-1 leukaemia inhibitory factor (LIF, *EuroClone*), 1 mM sodium pyruvate (*Invitrogen*), 0.1 mM non-essential amino acids (*Invitrogen*), 2 mM L-glutamine (*Invitrogen*), 0.1 mM 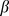-mercaptoethanol, and 500 U ml-1 penicillin/streptomycin (*Invitrogen*). RA was added as a DMSO (Dimethyl Sulfoxide) solution at a final concentration of 1,5 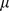
M to induce differentiation. Control cells were treated with an equal volume of DMSO. The cells were incubated at 37°C in 5% CO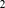; the medium was changed daily and the cells were routinely split every 2 to 3 days. Cells were then fixed in 4% PFA/PBS at 4°C overnight. After digestion with proteinase K, the cells were hybridized overnight with 1 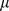
g digoxigenin–labeled riboprobe or biotin–labeled riboprobe at 60°C. The cells were then washed, blocked, incubated with alkaline phosphatase–conjugated anti digoxigenin antibody or streptavidin–AP conjugate, and incubated with NBT/BCIP detection buffer for 30 min. RNA probe preparation 200 ng of cDNA were PCR–amplified in 50 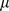
l PCRs using SP6 (5–GATTTAGGTGACACTATA–3) and T7 (5–TAATACGACTCACTATAGGGA–3) primers. PCR products were purified using a QIAquick PCR purification Kit (*Qiagen*), eluted in 30 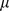
l of buffer, and quantitated using a NanoDrop. Digoxigenin–labeled RNA probes were transcribed from the PCR product templates using DIG RNA Labeling Kit (*Roche*) and the appropriate RNA polymerase. Probes were purified through RNA column and quantified by agarose gel electrophoresis or by running an RNA 6000 Nano Assay on a 2100 Bioanalyzer. Then 57 images were captured at 2560
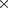
1920 pixel resolution in TIFF format at 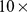 magnification in optical microscopy.

### Colony Segmentation

**Figure 2:**
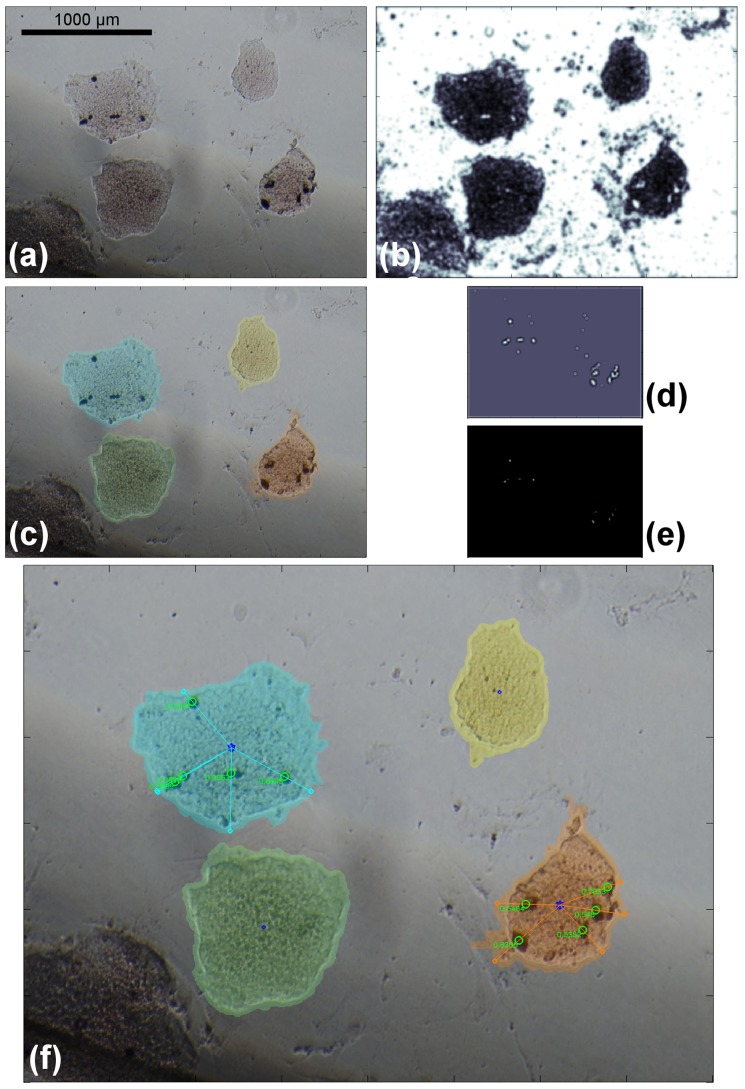
Segmentation process. (a): original image, (b): background subtraction. (c): colony segmentation, (d, e): orientation matching. (f): output image. Segmentation, identification and other outputs are shown overimposed on the original image (for a description see Figure 3).

Since we are interested in extracting cell locations inside the colonies, the first step of our pipeline is aimed at detecting colonies [13]. After a preprocessing step to remove the background and uniform the light intensity conditions, we apply a segmentation process where colonies in the image are segmented as disconnected single objects using a simplified two-dimensional version of our previously developed Enhanced Interaction Model [14]. The resulted binary image, containing the segmented colonies and the background, is then enhanced through a cascade of morphological operators. Different colonies are then processed through a Watershed transform which returns as output the segmentation for each colony in the image. The overall pipeline is depicted in Figure 1, while the various steps are described in detail below. We applied similar pattern analysis approaches in other biological domains, such as the identification of structural chromosome aberrations and carcinogenesis [15]. The main difference with the procedure depicted in Figure 1 is the use of a variational method to efficiently detect colonies and the setup of a statistical framework to test biological hypotheses relative to the expression behavior of genes of interest within Mouse ES Cells. The results of the various steps are reported in Figure 2.

#### Background Removal

The original image 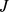 is passed through a morphological top hat filtering for background removal with a structuring element as a rolling ball 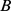 of radius 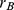 and height 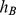. The image is then adjusted with a CLAHE algorithm [16], and the border intensity is enhanced and then blurred:
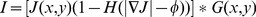
(1)where 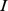 is our final filtered image, 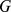 is a Gaussian filter of dimension 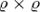 and standard deviation 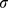, 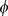 is a threshold and 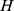 is the Heaviside step function, so that 
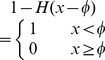
 and 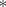 is the convolution operator. The results of the application of this process to a generic image is reported in panel (b) of Figure 2.

#### Segmentation

To segment the colonies we use a simplified, single contour, two-dimensional version of the Enhanced Interaction Model [17] presented in [14] where an energy functional 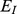 associated to the image is defined as
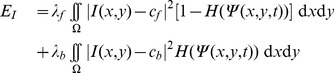
(2)


here 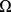 is the whole image domain, 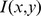 is the image to be segmented, 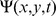 is a level set function [18], [19] whose zero level is the segmenting contour and whose negative levels represent the inside of the segmented object, 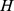 is the Heaviside step function as above, so that if 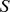 is the segmented shape its intern 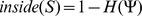, and so 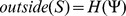, 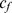 and 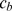 are the intensity means of the segmented foreground object and the background respectively, and 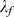 and 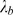 are weighting parameters. The evolution equation for 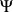 is then obtained by deducing the associated Euler-Lagrange equation:
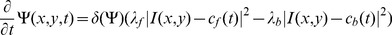
(3)where 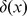 is the delta of Dirac, and 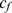 and 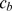 become functions of 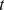:
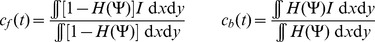
and their values must be updated accordingly. The results of the application of this process to a generic image is reported in panel (c) of Figure 2.

#### Binary Enhancement

We obtain a binary image 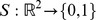 containing only the segmented shapes by simply calculating 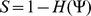. A dilation is performed in the image, defined as:
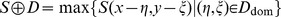
(4)where 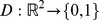 is a morphological structuring element with the shape of a disk with radius 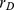 and where 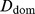 is its domain [20], [21]. Then the holes in the image are filled with a morphological reconstruction, intending by hole a background area that cannot be reached by filling in the background from the edge of the image domain [22]. Finally, all the connected components (objects) with very small areas are removed with a morphological opening. The unique segmenting contour 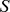 is then split into 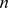 contours 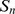, one for each colony in the image through a Watershed Transform [23].

### Cell Analysis

Identification of the marked cells inside each colony can now be performed: intra–colony stem cell location is in fact essential for the future organism formation [24]. A preprocessing step uses a Non-Linear Diffusion filtering to remove noise from the image with a smoothing process, though preserving the borders that are essential for the next spotted cell recognition step. Spotted cells are roughly circular, and a circular object recognition approach is needed to identify them. To achieve this, the filtered images are processed with an Orientation Matching algorithm that identifies circular and semi-circular objects within a range of desired radii. In order to perform the search for spotted cells, a preprocessing step is needed to soften the textures of the image and remove the noise; after that it is imperative that the borders of the objects are preserved: to do this a non linear diffusion operation is performed [25]–[27].

#### Orientation Matching

We use the approach proposed in [9] to identify spotted cells on the filtered image 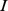. We first define the image gradient 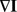 as
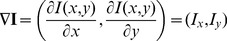
(5)


We also define an artificial gradient built in the form of an annulus 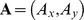 which is centered in 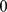 with radii 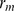 and 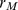 and having that each point in 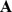 has the same gradient length and orientation pointing perpendicularly towards the annulus' edge. We now introduce the *Orientation Matching* function 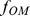


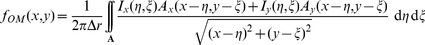
(6)where 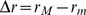 (for more details see [28]). In the implemented algorithm 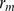 and 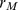 were not used in a single annulus but as extrema of smaller annuli of radii 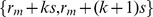 where 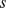 is a step value, 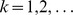 and 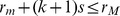.

To set (6) in a more suitable form we introduce the normalized gradient of 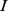


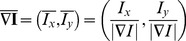
(7)and the normalized version of 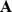


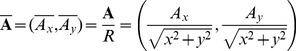
(8)where 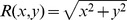 is the distance from the origin of each point inside the annulus (remember that 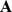 is centered in 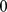). So the final formulation of 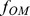 is



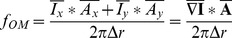
(9)Panels (d,e) of Figure 2 report a picture of the 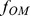 for the selected image.

### Location Analysis

Data from the identified spotted cells are then collected, relatively to each cell position inside the colony and to the other spotted cell positions. A hypothesis testing statistical approach [29] is then adopted to verify whether the marked cells have a preferred location behavior. Since standard randomness tests do not sufficiently take into account the biological problem, we perform a more restrictive location analysis by using a sampling approach that tries to model the underlying biological phenomenon to generate the null hypothesis. In particular, we randomly generate colonies to compute the sampling null distribution of descriptive location parameters such as the distance from the centroid, and the mutual distances between marked cells. The null distributions are then compared against the observed data with the non-parametric Kolmogorov-Smirnov test [30]. In order to drive biological conclusions we set the confidence level 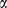 to be 0.001. Indeed, here we have a large biological variation; so that when the real dataset is small (as it was the case with one of our datasets) it cannot be expected that the curves from real and null cases will match closely even if the marked cells have a non-preferential localization. This is because statistically with a small number of data the observed distribution may differ slightly from the theoretical, real distribution [29]; so we chose to be more stringent with the confidence interval, and we then decided for a lower value.

The generation of the null distribution is performed in the following way: the real colonies are repopulated with points appearing randomly, whose distributions (distance from centroid and mutual distances) are then calculated. The points are generated by calculating the bounding box of the colony and randomly generate a 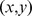 coordinate pair inside the box; the points falling outside the colony are then discarded. For each colony the points generated in the null case are ten times the number of real points.

Before testing the hypotheses the segmented colonies are normalized, i.e. transformed into a circular shape with unitary radius; indeed, real colonies are semi-circular but not perfect circles. Such transformation then keeps into account the distance from the centroid and at the same time from the colony's edge. Let's define as 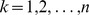 the number of each spotted cell into the colony, as 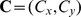 the colony centroid and as 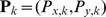 the position of the cell 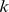. We can now define the distance of the cell 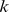 from the centroid:
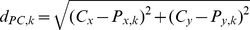
(10)


Let's now define 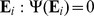 i.e. 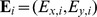 are all the points on the edge of the colony. We are interested in the edge point 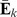 which lies on the same semirect passing through 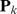 and originating from 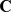. To find 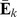 we have to calculate
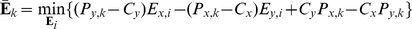
(11)


The distance from the centroid 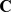 and 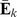 is then
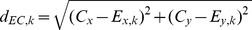
(12)


Now the normalized distance of the point 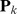 (referred to a unitary circle, see above) is calculated as
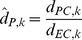
(13)so that 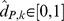. The next step is to compare the distribution of the null case with that of the real case: we used the non-parametrical Kolmogorov-Smirnov test [30] to compare the real distances with the *in silico* one.

Following the Kolmogorov-Smirnov analysis, a direct classification of the marked cells can be performed. If the colonies expressing the gene of interest show that the location of the gene-expressing cells is not statistically different from that of the null case, its location pattern may be labeled as NON-PREFERENTIAL; otherwise they can be classified as PREFERENTIAL since they have a preferential intra-colony location. The difference from the null case can be studied by the real case data distribution, for example showing if they are closer to the centroid or to the edge by comparing the means, obtaining the sub-labels INNER and OUTER.

Clusterization of the marked cells is also valuable information that may lead to precise biological hypotheses. In most cases it may be deduced from a non-uniform intra-colony location, but we tested it quantitatively. For each colony the mutual distances between cells were calculated as
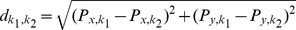
(14)for every possible pair of cells 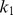 and 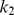. They were then normalized in 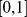 by dividing for the major axis of the colony
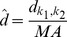
(15)where 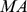 is the major axis. Distribution of mutual distances in the real cases are then compared to null cases; the overlapping of the distribution curves implies a non clusterization, while the opposite means that marked cells appear in definite groups. This adds the sub-labels CLUSTERED and NON-CLUSTERED.

### Application

A MATLAB script pack which implements the proposed method and is capable of a full automated analysis has been developed and it is available at http://bioinformatics.biogem.it/, together with the images reported in the Results Section. A flow diagram of the proposed approach is shown in Figure 1 and the results of the various steps are reported in Figure 2. All the adopted parameters of the procedure, which were used in the experiments, are reported in Table 1.

**Table 1:** Table of the parameters adopted for the analysis.

	*Zscan4*	*EndoA*	*Nanog*	*Pou5f1*
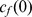	0	0	0	0
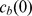	255	255	255	255
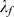	1.0	1.0	1.0	1.0
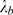	1.0	1.0	1.0	1.0
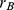	12.0	12.0	12.0	12.0
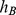	4.0	4.0	4.0	4.0
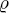	25.0	25.0	25.0	25.0
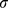	2.5	2.5	2.5	2.5
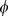	0.75	0.75	0.75	0.75
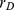	5.0	5.0	5.0	5.0
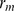	6	4	10	10
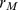	9	10	25	24
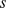	1	1	2	2
magnif.	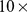	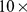	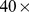	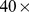
resize	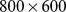	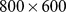	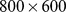	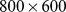

## Results

**Figure 3:**
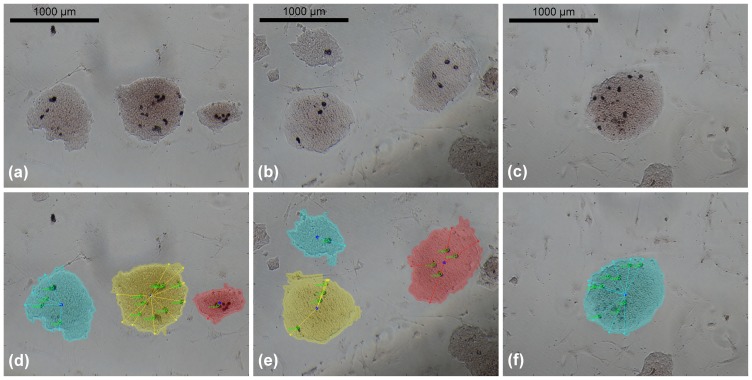
Segmentation examples. (a, b, c): original images. (d, e, f): segmented colonies and identified spotted cells. Colonies are segmented with different colors: in each one the central blue star point is the centroid while small green circles identify marked cells; the segment from the centroid to the marked cell defines the normalization path, whose value is shown next to the small circle.

We proposed a method capable of 1) automatically segmenting ES colonies and identifying marked cells of interest, and 2) extracting quantitative location data and performing statistical analyses which can lead to biological hypotheses about the cells of interest behavior (see Figure 3 for examples of segmentation results). We implemented the proposed approach in MATLAB and tested it on a set of 57 optical microscopy images obtained from culture of ESCs followed by *in situ* hybridization. Images were 2560
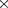
1920 pixel resolution in uncompressed tiff format at 10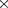 magnification, acquired with a Zeiss microscope with 0.94 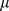
m pixel resolution. Because the proposed algorithm becomes computationally demanding on large images, they were resized at 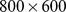 pixel resolution during analysis to achieve better performances. This standardization also allows to use the same parameter settings for all the reported experiments. The colony segmentation tended to extent a little outside the real colony edges; to overcome this the distances in the interval 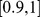 (i.e. at the extremities of edges) were truncated. Also, due to the limited number of images, a slight smoothing was performed on the distribution curves to overcome individual case peaks in the resulting distribution, using a moving average with windows size 3.

Our first aim was to validate the performance of the cell detection method based on the Orientation Matching Transform, explained in Materials and Methods, in terms of precision and recall. In particular we manually collected a true table for a set of images containing 903 marked cells. Our procedure resulted in 676 true positives, 227 false negatives and 8 false positives. Hence we have a precision of 98.8% and a recall of 74.9%. Within this context we are more interested in the rate of type I error (false positives), since it can significantly alter the biological conclusions, whereas the rate of false negatives is less relevant, since with a sufficient number of samples the same conclusion could be derived. Indeed, even if some marked cells are not detected, the effect can be similar at having less images or samples; however when the amount of detected cells (i.e. the data effectively collected) is enough to draw conclusions at the chosen significance level, we can reliably derive location preferences. In our case we used a two-sample Kolmogorov-Smirnov test which requires a number of samples in order of few hundreds [29] so our detected cell number can be considered adequate for the statistical analysis we are performing. For those reason we tuned the Orientation Matching parameters in order to reduce the risk of type I errors; in conclusion we consider the cell detection accuracy appropriate for the biological question we are posing.

### Location analysis of *Zscan4*

*Zscan4* was an elite candidate gene because it marks a subpopulation of ESCs – defined as mosaic-in-colony cells – in regular culture condition on whose expression behavior the analysis was carried out. No transcripts of the *Zscan4* family are detected in any cell types other than ESCs, thus being an ideal gene to be studied under morphogenetic conditions. The expression of *Zscan4* starts during the first wave of transcription, called zygotic genome activation (ZGA), and begins during the 2-cell stage in mouse preimplantation development and marks a vital transition from the maternal to the embryonic genetic program. Preparation of the images of *Zscan4* is described in detail in the Materials and Methods section.

**Figure 4:**
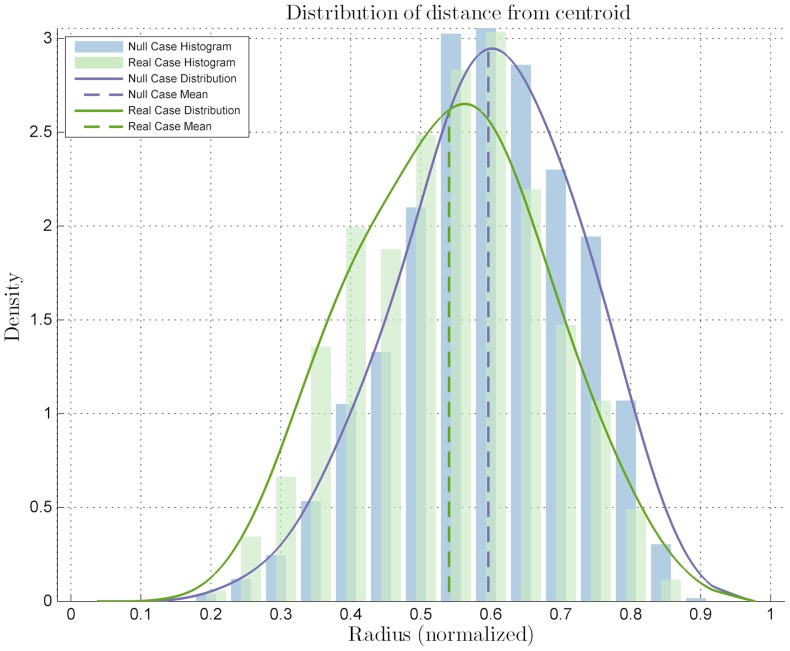
Distribution for the statistical analysis on the *Zscan4*-marked ES colony image set. Distribution related to the distances from the centroid compared to the null case distribution, with a statistical difference *p-value* of 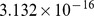 with significance level 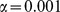.

**Figure 5:**
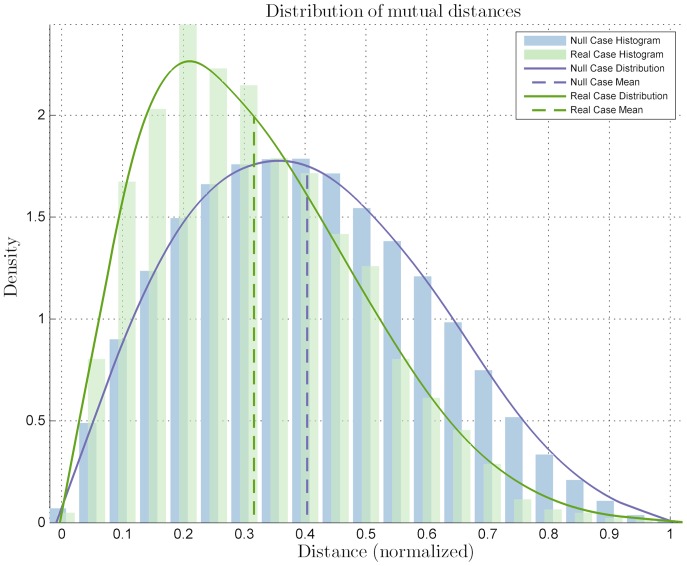
Diagrams for the statistical analysis on the *Zscan4*-marked cells mutual distances. Distribution related to the mutual distances compared to the null case distribution, with a *p-value* of 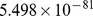 with significance level 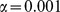.

The images were processed with the proposed algorithm, resulting that the cells expressing *Zscan4* did not fit the null case distribution, thus having a location preference and being classified as preferential (Figure 4). Immediate observation of the mean on the 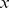 axis location also suggested the sub-labeling of inner. The distribution of the distances 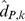 was different from that of the null case, with a *p-value* of 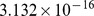 from the Kolmogorov-Smirnov test. The distribution mean is located closer to the center of the colony than the null case mean, thus suggesting a more central location of the cells expressing *Zscan4*. Also, clusterization of the marked cells among themselves may be postulated because of the same number of cells appearing more centrally in the colony, where there is less space. This observation was confirmed by the clusterization quantitative analysis: the mutual distance 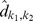 distribution shows a significant difference with that of the null case (Figure 5) with a *p-value* of 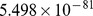. This sub-labels *Zscan4*'s behavior as CLUSTERED. The significance level for all the statistical tests was set as 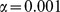.

### Control Genes

Three other genes were selected as controls, *EndoA* (or *Lrrk2*), *Nanog* and *Pou5f1* (or *Oct4*). They all have known behaviors and they have thus been chosen as control genes to test the proposed pipeline: *EndoA* is known to be a differentiation marker and is known to be expressed only on the edge of the colony [31]. *Nanog* is a marker of metastability for the ESCs, it is thus expressed without a preferential location inside the colony [32] and *Pou5f1* is also a metastability marker: its expression must be closely regulated, causing otherwise differentiation inside the colony [33].

**Figure 6:**
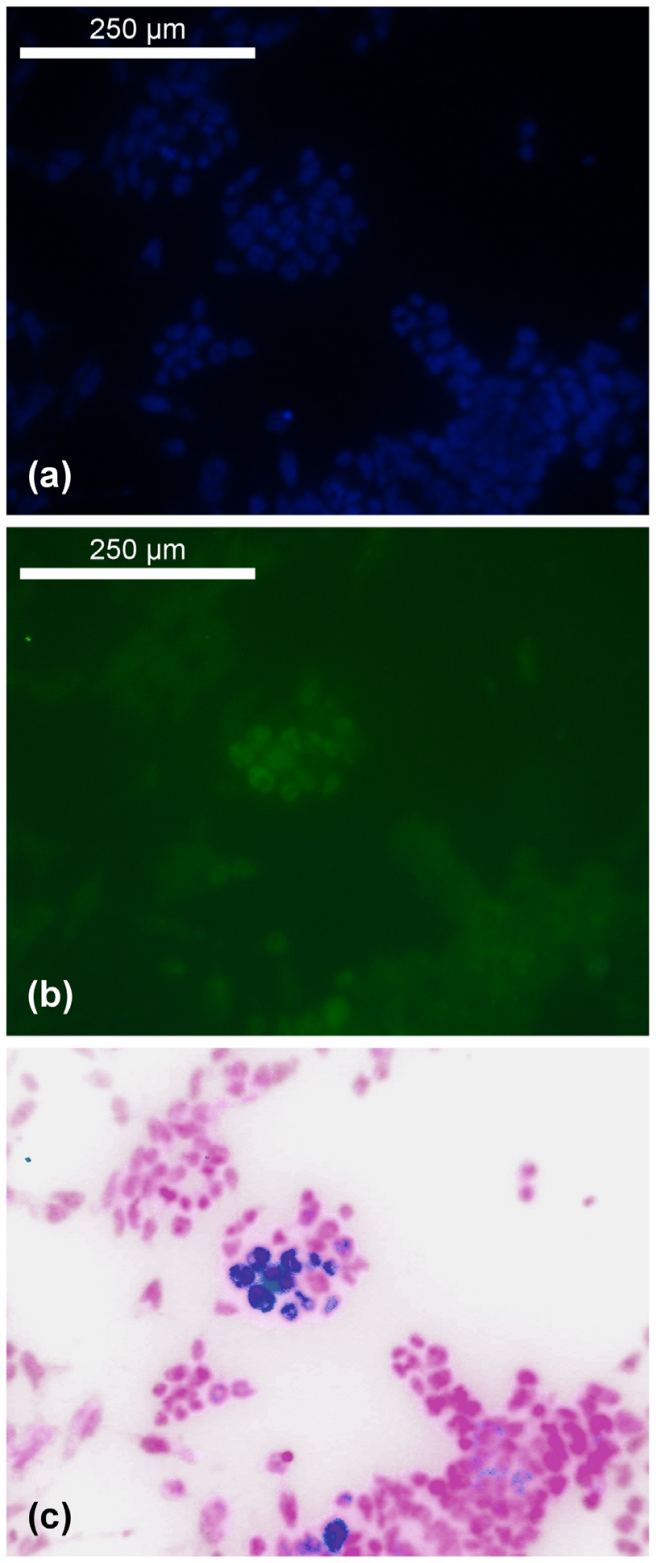
Fluorescence images. Union processing of two channels for the fluorescence microscopy images (this is from the 40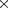 *nanog*-marked colony image set). (a): DAPI channel (blue), (b): *Nanog* channel (green), (c): resulting image; it was preprocessed so the colors were similar to those in the *Zscan4* set so that the algorithm could be applied immediately.

The *EndoA*-marked set was composed of 10 optical microscopy images obtained from cultured ESCs followed with *in situ* hybridization. Images were 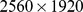 pixel resolution in uncompressed tiff format at 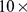 magnification. The *Nanog*-marked and *Pou5f1*-marked sets were composed of 30 fluorescence microscopy images each, showing cultured ESCs followed by *in situ* hybridization. Images were 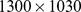 pixel resolution in best quality jpeg format at 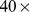 magnification. There were two fluorescence channels: a DAPI channel for nuclei marking and the specific fluorophore for the protein codified by the desired gene. The two fluorescence channels were manually combined together to have a single optical-like image to be passed to the algorithm (see Figure 6), by merging the images and shifting the hues of the channels. By doing so the images could be immediately passed to the algorithm without much further parameter tuning. It has to be noted that *Nanog* and *Pou5f1* are peculiarly expressed in undifferentiated ESCs, i.e. they are expressed everywhere inside undifferentiated colonies.

The light field microscopy of *Zscan4* experiments represented RNA detection through *in situ* hybridization assay. We could not perform immunofluorescence microscopy because there is no commercial antibody available. We tried to detect *Nanog* and *Pou5f1* RNA signals through *in situ* hybridization but unfortunately their RNA expressions were too weak to be detected by the sensitivity of this technique. At this point we relied on a more sensitive detection assay such as immunofluorescence of *Nanog* and *Pou5f1* proteins using commercial antibobies. Being capable to use two detection assays based on RNA and protein respectively, and having our results to be consistent between them, we can conclude that our algorithm is general and flexible and thus not technique-dependent.

**Figure 7:**
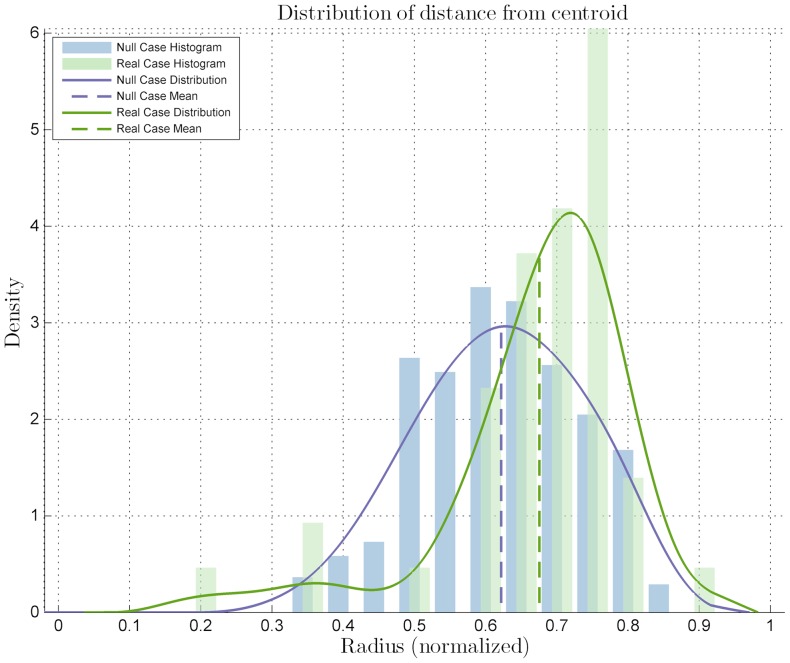
Diagrams for the statistical analysis on the *EndoA*-marked ES colony image set. Distribution related to the distances from the centroid compared to the null case distribution, with a statistical difference *p-value* of 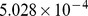 with significance level 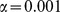.

**Figure 8:**
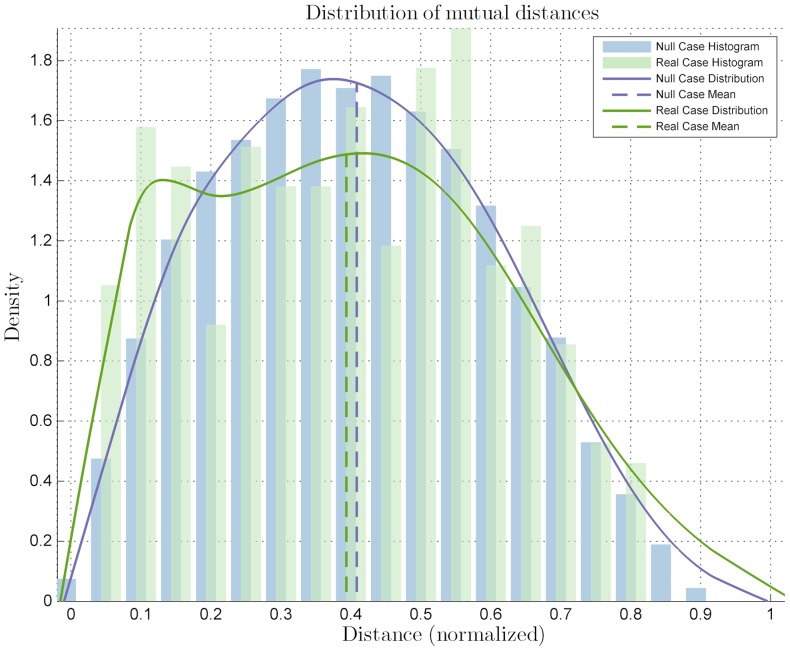
Diagrams for the statistical analysis on the *EndoA*-marked cell mutual distances. Distribution related to the mutual distances compared to the null case distribution, with a *p-value* of 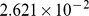 with significance level 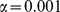.

**Figure 9:**
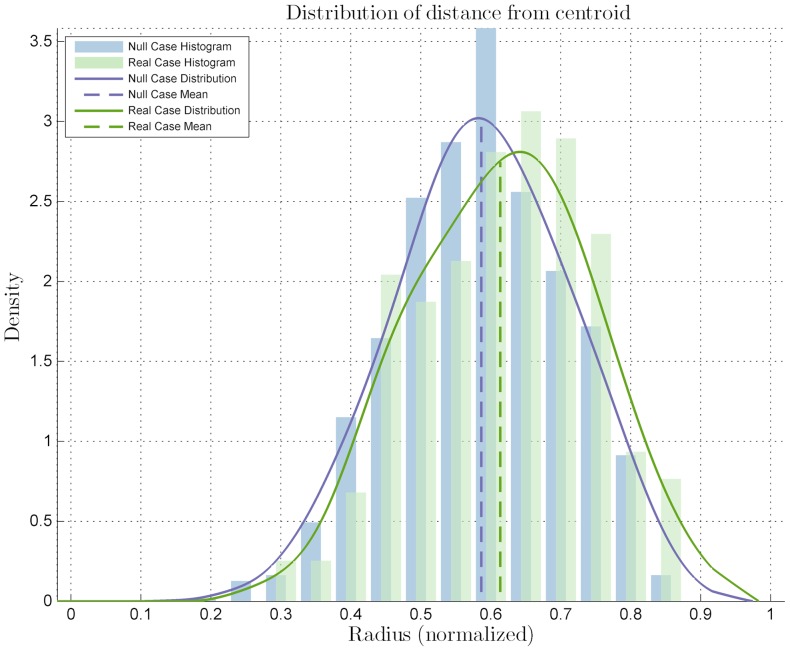
Diagrams for the statistical analysis on the *Nanog*-marked ES colony image set. Distribution related to the distances from the centroid compared to the null case distribution, with a statistical difference *p-value* of 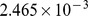 with significance level 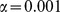.

**Figure 10:**
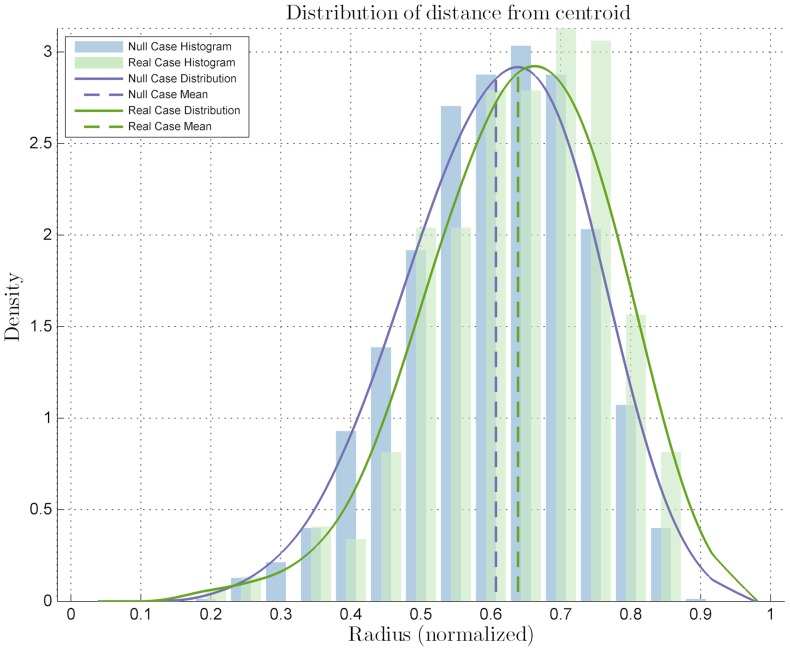
Diagrams for the statistical analysis on the *Pou5f1*-marked ES colony image set. Distribution related to the distances from the centroid compared to the null case distribution, with a statistical difference *p-value* of 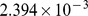 with significance level 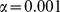.

As expected, the results for *EndoA* (Figure 7) show that the marked cells distribution is classified as PREFERENTIAL (*p-value* 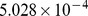), and the mean is also located towards the colony edge, thus they are also sub-labeled as OUTER. Moreover, they have no clusterization behavior, which is confirmed by the quantitative mutual distance analysis that shows a distribution very similar to the null case (Figure 8) with a *p-value* 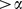; this sub-labels them as NON-CLUSTERED. The results about *Nanog* (Figure 9) show that its behavior is basically NON-PREFERENTIAL (*p-value* 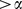); the same holds for *Pou5f1*, where the results are very similar (Figure 10) showing a lack of location preference as behavior of the marked cells.

For these two genes a quantitative analysis of clusterization revealed, as expected, that they are NON-CLUSTERED with *p-values* 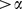 indeed, in these two cases those genes are expressed everywhere in the colony and therefore marked cells appear very dense, forming *de facto* a unique large cluster spread throughout the whole colony.

## Discussion

We presented a novel algorithm capable of automatically identifying the location of cells expressing a gene of interest into stem cell colonies and of executing automatic quantitative measurements followed by a statistical analysis. We tested the model on the *Zscan4* gene, showing that it has a preferential location behavior into the colonies and is preferably located towards the colony centroid, so that the cells expressing *Zscan4* tend to be clustered; all measurement were compared to a completely location preference lacking *in silico* model.

Functionality and reliability of the proposed approach were tested on three control genes, whose behavior is well-known: *EndoA*, *Nanog*, and *Pou5f1*. The analyses showed that the results were concordant with the expected behavior of those genes, thus assessing that results from our approach are trustworthy. This is of great importance because it allows us to put up biological hypotheses about the role of *Zscan4* on morphogenesis: first we can state that *Zscan4* is not expressed inside a stem cell colony without a location preference, and that it is instead somewhat bound to the internal sectors of the colony. In addition to the above, *Zscan4*-expressing cells are also clustered between themselves; this is the most notable aspect about their appearance behavior and may be related to morphogens (controlling or controlled through the expression of *Zscan4*) that are diffused in the morphogenetic process.

Similiar analysis may of course be carried out on other genes of interest, enlightening location behavior of the cells expressing it, thus leading to important clues in understanding their role in the morphogenetic process of higher organisms.
